# A Dilute and Shoot Strategy for Determining *Alternaria* Toxins in Tomato-Based Samples and in Different Flours Using LC-IDMS Separation

**DOI:** 10.3390/molecules26041017

**Published:** 2021-02-15

**Authors:** Ádám Tölgyesi, Tamás Farkas, Mária Bálint, Thomas J. McDonald, Virender K. Sharma

**Affiliations:** 1 Bálint Analitika Ltd., Fehérvári út 144, 1116 Budapest, Hungary; farkas.tomi.ft@gmail.com (T.F.); titkarsag@balintanalitika.hu (M.B.); 2 Program for the Environment and Sustainability, Department of Environmental and Occupational Health, School of Public Health, Texas A&M University, 212 Adriance Lab Rd., 1266 TAMU, College Station, TX 77843, USA; t-mcdonald12332@tamu.edu

**Keywords:** *Alternaria* toxins, LC-MS/MS, isotope dilution, dilute and shoot, food samples

## Abstract

*Alternaria* toxins are emerging mycotoxins whose regulation and standardization are in progress by the European Commission and the European Committee for Standardization. This paper describes a dilute and shoot approach to determine five *Alternaria* toxins in selected food samples using liquid chromatography–tandem mass spectrometry (LC-MS/MS). The strategy involves sample extraction with acidified aqueous methanol, followed by a solvent change accomplished via sample evaporation and reconstitution. The quantification is based on isotope dilution, applying all corresponding isotopically labeled internal standards to compensate possible matrix effects of the analysis. The main advantages of the present method over other existing methods includes simple and effective sample preparation, as well as detection with high sensitivity. The five-fold sample dilution can decrease matrix effects, which were evaluated with both external and internal standard methods. The results demonstrated a limit of quantification lower than 1.0 µg/kg for all five analytes for the first time. The newly presented method showed acceptable accuracy (52.7–111%) when analyzing naturally contaminated and spiked standard samples at the described levels. The method was validated for tomato-based and flour samples (wheat, rye, and maize). The absolute recovery ranged from 66.7% to 91.6% (RSD < 10%). The developed method could be an alternative approach for those laboratories that exclude sample cleanup and pre-concentration of state-of-the-art instruments with enhanced sensitivity.

## 1. Introduction

In the last 10 years, many natural food contaminants have emerged, such as ergot alkaloids, tropane alkaloids, phomopsin A, *Alternaria* toxins, and citrinin, all of which are of great interest to the global scientific community [1,2,3,4,5]. Generally, toxins are stable, having toxic effects causing potentially chronic and acute diseases [6,7]. Therefore, the maximum levels (MLs) of some of these food contaminants have recently been set or are under consideration by the European Commission (EC) [8,9]. For example, the European Food Safety Agency (EFSA) has already published scientific opinions on these contaminants, indicating the importance of food contamination to population health [1,2,3,4,5]. 

Analytical methods to quantify emerging food contaminants are either standardized by the European Committee for Standardization (CEN) or have already been published by the CEN [10,11,12,13]. The primary focus is on the *Alternaria* toxins and secondary metabolites (mycotoxins) that are produced by *Alternaria alternata* growing on agricultural commodities [4]. According to the EFSA, more than 70 *Alternaria* toxins have been identified. Of these toxins, alternariol (AOH), alternariol monomethyl ether (AME), and tenuazonic acid (TEA) have preliminary MLs [9]. Currently, the MLs are not regulated, yet these levels may indicate the potential concentration levels of concern in food. The lowest levels of concern (5 µg/kg and 10 µg/kg) are those considered for AME and AOH, respectively [9]. The most highly infected foods are vegetables, cereals, and sunflower seeds. 

The mandate for standardizing *Alternaria* toxins in foods was given to the EC Joint Research Centre (JRC, Geel, Belgium) by the CEN [10,14]. The JRC organized inter-laboratory validations for these toxins involving tomato juice, puree, cereals (e.g., wheat, sorghum, triticale), and sunflower seeds (both peeled and unpeeled) [10,13]. The concentration levels set for validation were 1–10 µg/kg for AOH and AME, and 10–1000 µg/kg for TEA [10]. Two other toxins, i.e., altenuene (ALT) and tentoxin (TEN), were also included in the standardization and validation study [10,13]. These toxins can rarely be found in food and, therefore, are not yet included in the preliminary planned MLs. However, the standard method, on the request of CEN, requires simultaneous analysis of ALT and TEN together with the analysis of the other *Alternaria* toxins, as mentioned above [10,13].

The recent literature on *Alternaria* toxins has shown that the most suitable technique to analyze *Alternaria* toxins in food is liquid chromatography–tandem mass spectrometry (LC-MS/MS) [6,13,14,15,16,17,18,19,20,21,22,23,24,25,26,27,28,29,30,31,32,33,34,35]. The research articles and inhouse validation of the candidate standard method focused on the significant matrix effect (ME) [13,14], which greatly influences the LC-MS/MS analysis of *Alternaria* toxins in food matrices [13,14,15,16,17,18,19,20,21,22,23,24,25,26,27,28,29,30,31,32,33,34,35,36]. Consequently, isotope dilution mass spectrometry (IDMS) must be employed in order to compensate for ion suppression, which is caused by co-eluting matrix constituents [13,18,20,24,25,27,30,31,35,36]. When the first trial was carried out for *Alternaria* toxins by the JRC, the stable isotopically labeled internal standards (ISTD) were not available in the market, and an approach of matrix-matched calibration was suggested [10]. However, this evaluation resulted in low reproducibility for AME and TEA analyses, which had been strongly influenced by the co-eluting matrix solutes. Currently, ISTDs are commercially available and the LC-IDMS method can now be carried out for to analyze *Alternaria* toxins. Further, the apparent compensation of the ME can also be done [13,18,21,24,25,27,30,31,35]. It is important to emphasize that all corresponding ISTD should be used because the different *Alternaria* toxins undergo various ME’s due to the different chemical structures and retention times in HPLC separation [13,18,21,24,25,27,30,31,35]. 

The effect of matrix constituents on ionization can be minimized by applying appropriate sample purification (see Table S1). Solid phase extraction (SPE) is generally used for extracting *Alternaria* toxins from food samples [13,14,21,26,29,33,34,35,36]. Hydrophilic modified reversed-phase polymeric cartridges (i.e., Strata-XL, Oasis HLB) have shown good retention for these toxins due to their medium polar or non-polar character (log*P* = 0.9–3.3) [10,13,16,31]. More efficient cleanup could be done to employ anion exchange cartridges due to the weak acidic character of the toxins (p*K*a = 4.3–7.7) [31]. However, TEN is a neutral molecule, so it has no retention on an anion exchange cartridge. Another aim of SPE is to avoid sample dilution during sample preparation; therefore, the low concentration levels (down to 1 µg/kg) can be accurately measured using LC-MS/MS instruments with enhanced sensitivity. Some studies recently suggested the quick, easy, cheap, effective, rugged, and safe (QuEChERS) sample preparation to analyze *Alternaria* toxins in various food samples [22,23,24,30]. This extraction method is generally used to analyze pesticides, so the raw food extract, prepared for toxins, can also be analyzed for pesticides. 

On the other hand, the recently launched instruments with high sensitivity may allow for the elimination of sample cleanup and pre-concentration, thus enabling the application of sample dilution prior to analysis. These kinds of methods would lead to the approach called the “dilute and shoot” strategy, which is generally used to analyze mycotoxins [37,38]. The instrument used in such an analysis should detect at least 10-fold lower mass concentration in a neat solution (*y*) than the lowest targeted mass fraction of analytes in the sample (*w*). The present study aimed to eliminate SPE cleanup by applying a dilute and shoot approach to analyze *Alternaria* toxins in food matrices. Recently, we put effort into developing dilute and shoot methods in order to analyze *Alternaia* toxins. The first approach involved sunflower oil samples [31]. The objective of the present study was to continue this research and to extend the method to other food matrices such as tomato-based samples and different flours using high sensitivity LC-IDMS separation. 

## 2. Results

### 2.1. Analysis of Quality Control Samples Using the Optimized Method

After the optimization of ion transitions (Table 1), ion source parameters, and HPLC separation (Section 4.3), the instrumental limit of quantifications (LOQs) in the neat solvent expressed in mass concentration were as follows: AOH- 0.01 ng/mL, AME- 0.001 ng/mL, TEA- 0.10 ng/mL, TEN- 0.01 ng/mL, and ALT- 0.10 ng/mL. At these concentrations, the signal-to-noise ratios (SNR) were above 10 for all ion traces, and the ion ratios were within the permitted tolerance limits (PTL) (Table 1). The improved sensitivity was also due to the use of a fused core HPLC column, which resulted in narrow peaks for toxins [20]. Therefore, the lowest recommended level in food (1 µg/kg for AOH, AME, and ALT) was CEN and the lowest natural contamination of AME (0.55 µg/kg) in wheat quality check (QC) sample was detected even after a five-fold sample dilution. This was confirmed by analyzing several (quality control) QC and validation samples. 

Naturally contaminated and spiked QC samples were tested using the method described in Section 4.2 and Section 4.3. A single analysis was carried out as requested in the trials [10,13]. The results revealed that all toxins could be detected in samples (Table 2) and most of the values were within the reference values ± standard deviation. The satisfactory range is the reference value ± 2.8 times the standard deviation (reproducibility limit) that was fully achieved. In the trial, the concentrations of AME and AOH (0.55 µg/kg and 2.04 µg/kg) in sample B (naturally contaminated wheat) were reported by only a few laboratories (Table 2) due to the low concentrations and high matrix complexity of this particular sample [13]. However, we could also detect that these concentration levels used our study’s described dilute and shoot method. It is clearly seen that the naturally contaminated wheat showed very different trueness values for the analytes. Additionally, different trueness was observed between the two wheat samples. The trueness may vary between analytes due to their different extractability from the wheat matrix, which could possibly be caused by the different chemical structure and hydrophobicity of the target analytes. Sample B was of a much different wheat than the other wheat sample and its extract was more complex, which did not allow for a full evaluation of AME and AOH in the trial. The trueness obtained are in accordance with the criteria (50–120%), which are recommended in the CEN/TR 16059 standard [39].

### 2.2. Method Performance Characteristics

The absolute and relative MEs were evaluated by analyzing three different tomato-based samples (tomato puree, ketchup, and tomato sauce) and three different flour samples (wheat, rye, and maize) [40]. No contaminants were found in the flour samples while tomato-based samples contained AOH and AME below 0.50 µg/kg. Further, the mass fraction of TEA was never higher than 10.0 µg/kg, while TEN and ALT were not detected in any of the tomato-based samples. Samples were prepared with six replicates and were post-spiked to obtain matrix-matched calibration samples. One sample from each was used as a blank to correct the responses obtained in spikes, and the other five sample extracts were post-spiked to obtain a five-point calibration. The levels of AOH, AME, and ALT were in a range of 0.5 to 10 µg/kg. In the case of TEA and TEN, the concentration ranges were 5.0–100 µg/kg and 2.5–50 µg/kg, respectively. The spiking level of TEN was lower than TEA due to the higher sensitivity of TEN.

The slopes of the matrix-matched calibrations (three for each matrix) were compared to the slope of calibrations prepared in the pure solvent used to evaluate the absolute matrix effect [40]. Results were calculated using both the ESTD (external standard) and ISTD (internal standard) methods (Table 3). The RSD% of slopes of matrix-matched calibrations for each matrix (tomato-based and flour) were used to calculate the relative matrix effect [40]. When ESTD was used for the evaluation, a considerable absolute ME% was found in flour matrices for each compound with the exception of TEA. The relative ME was not higher than 10.4% in flour samples, so the matrix-matched calibration might be used for the compensation of the matrix effect, but it is not recommended. The ME was well compensated with ISTD; both absolute and relative ME’s improved with IDMS. The evaluation with the ESTD method demonstrated that the different compounds underwent various ME’s. Therefore, the corresponding ISTDs must be used for all analytes [31]. The ME’s were lower in tomato-based samples, but AOH was considerably affected by the background in this matrix (Table 3) as well. A moderate ME influenced the analysis of toxins in tomato-based products, except for AOH. The sample dilution performed during sample preparation should result in reduced MEs in this matrix. The ME was improved for AOH with IDMS; however, the IDMS did not significantly improve the MEs for the other compounds. This suggests that the sample dilution already reduced the ME. Generally, AOH, AME, and TEA have high MEs [13,18,20,24,25,30,31,32,33,36]. However, only the signal of AOH was considerably influenced by MEs in all matrices analyzed using the described method. Another important conclusion of the ME study was that the reduced ME did not increase the LOQs in tomato-based samples.

The method was validated with tomato-based samples and flour samples. The spiking levels were 2.0 µg/kg and 10.0 µg/kg for AOH, AME, and ALT. In the case of TEA and TEN, the fortification levels of 40 µg/kg and 200 µg/kg (Table 3) were used according to the method validation study (MVS), performed in 2015 and 2018 [10,13]. In total, 10 tomato-based samples (four tomato purees, three ketchups, and three tomato sauces) and flour samples (four wheat, three ryes, and three maize) were prepared for both matrices at each level over two days and analyzed to calculate the absolute recovery and precision. The recoveries were between 50.0% and 120%, meeting the previously referenced criteria [39]. The within laboratory precision did not exceed 10% (Table 3), which also met the standard [39]. 

**Figure 1:**
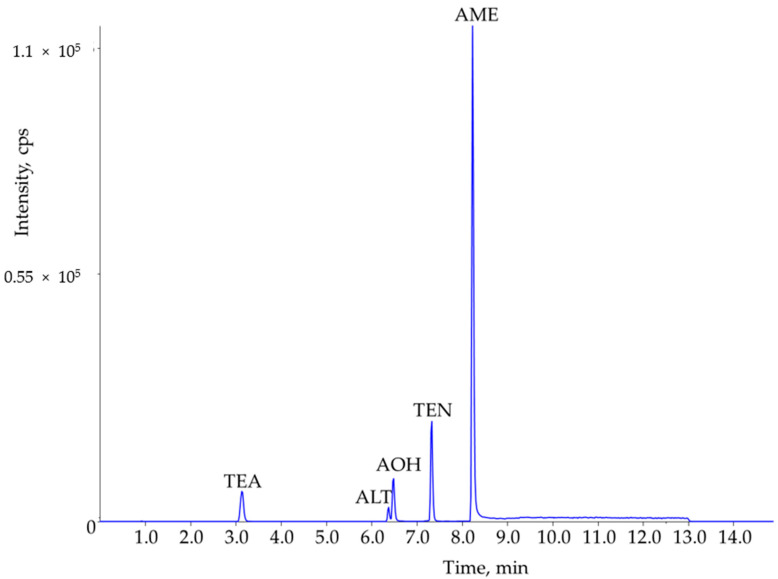
Total ion chromatogram of a tomato juice sample spiked with AOH, AME, and ALT in 2.0 µg/kg concentration and with TEA and TEN in 40 µg/kg concentration.

The selectivity was evaluated by comparing chromatograms of blank and fortified samples (Figure 1). Interfering peaks did not disrupt the analysis. However, a matrix peak did elute close to the peak of ALT-d6 on the more intense ion trace (296.1 > 217.1 *m/z*). This matrix peak had a high response in ketchup, and thus lowered the accuracy based on ISTD evaluation. Therefore, the less sensitive ion trace of ALT-d6 (296.1 > 189.1 *m/z*) was used (Table 1), which was free of an interfering peak. The identification was based on the retention times and the ion ratios that were within the tolerance limits (average ion ratio in solvent ± 30%). The LOQs in the sample matrices were evaluated based on the SNR calculated on both ion traces, and the ion ratios were also considered. The LOQs were set as SNR > 10 at all ion transitions and the ion ratios were within the permitted tolerance limits (Table 1). The high sensitivity of MS/MS detection and the reduced matrix suppression allowed the LOQs to lower to 0.02 µg/kg for AME in tomato-based samples. The LOQ of AOH was at least five times lower than the lowest validation level (1 µg/kg) in the MVS in 2015 and 2018 [10,13]. The highest LOQs (0.70 µg/kg and 0.80 µg/kg) were calculated for TEA and ALT in flour samples (Table 3). 

## 3. Discussion

### 3.1. LC–MS/MS Analysis of Alternaria Toxins 

Research articles dealing with *Alternaria* toxin analysis in various food samples using LC–MS/MS have been frequently published over the last 10 years. Earlier papers reported the determination of a couple of analytes (i.e., AOH and AME or only TEA) (Table S1), but more recent methods involve multiple compounds, with few of them included in the masked *Alternaria* toxins [20,24,25,36]. In our study, we focused on those five toxins, which are specified for standardization [10]. It is expected that the regulated MLs of the toxins will be set in the near future. Recently published studies clearly indicate that accurate LC–MS/MS determinations for *Alternaria* toxins could be carried out using IDMS for quantification [13,18,21,24,25,27,30,31,35], but only a few papers involved all isotopically labeled analogs in the method [13,18,27,30,31,35]. This is quite important because the investigation of ME (Section 2.2) highlighted that the corresponding isotopically labeled ISTD should only be used for a specified compound. Otherwise, the ISTD and the target compound will undergo various MEs and the accuracy will be lower. 

The aim of this study was to use a dilute and shoot approach in conjunction with the IDMS to analyze *Alternaria* toxins in selected food samples, which are commonly infected with toxins. The dilute and shoot method used to analyze mycotoxins have been known for a while [38], but the *Alternaria* toxins mentioned above are not included in these dilute and shoot methods, since they require special HPLC conditions and unique extraction solvents [10,13]. TEA has irreproducible HPLC behavior under acidic pH conditions, and, therefore, the HPLC separation of TEA at alkaline pH has been devised for a pre–column derivatization where 2,4–dinitrophenylhydrazine (Table S1) must be performed. Both approaches allow the simultaneous analysis of *Alternaria* toxins (Table S1); however, the dilute and shoot approach excludes derivatization, and so we applied alkaline pH in the LC–MS/MS method. The alkaline pH conditions improved the sensitivity in the ESI negative ion mode. Moreover, the sensitivity was further enhanced using a fused core HPLC column for LC separation [20]. This column established narrow peaks for the compounds and improved the SNR accordingly.

In contrast to other mycotoxins, *Alternaria* toxins are preferably analyzed with a methanolic–based extraction [10,14,31] because they are more soluble in methanol and only slightly in acetonitrile [41]. However, we also reported acetonitrile–based extractions (Table S1). The methanolic–based extraction used was optimized earlier [10] to achieve the best accuracy, which was further enhanced by sample dilution and IDMS. The tomato–based aqueous samples were extracted with pure methanol [14]. In the case of cereals, the extraction medium should involve water to let the starch swell, and then any mycotoxins that might be trapped inside the starch network could become highly extractable. This could be further improved by an acidic medium. The high methanolic extraction was necessary due to the lipophilic character of AOH and AME (logP = 3.2–3.3). The five–fold sample dilution could be very important because high MEs influence the analysis of Alternaria toxins [13,14,18,21,24,25,27,30,31,35,36]. The previous methods may pre–concentrate on co–eluting matrix constituents together with the target compounds during reversed–phase SPE cleanup (Table S1).

Another important aspect of the quantification of toxins is the application or exclusion of SPE cleanup. AME has high sensitivity in MS/MS detection, employing negative ionization. However, AOH and ALT have less sensitivity, and therefore sample pre–concentration should be done (Table S1). The highest LOQ can typically be achieved for ALT. In our study, an instrumental LOQ of 0.10 ng/mL in a pure solvent could be achieved after fine–tuning the detection parameters and using a fused core HPLC column. The optimization of ion transitions were simply carried out with the compound optimization software of Analyst, and ion traces implemented were those that had the highest abundance, except for ALT–d6 (Section 2.2). The ion source parameters (i.e., gas flows and temperature) were adjusted to the LC flow, and those optimal parameters are described in Section 4.3. A gas temperature higher than 350 °C did not increase the response.

It should be mentioned that alkaline pH conditions for HPLC separation are not only important for TEA but also for ALT because acidic pH can suppress ALT ionization, thus lowering the sensitivity to its detection [14]. The application of SPE, preferably a hydrophilic modified polymer, enables the purification of the sample from the hydrophilic matrices that elute at the beginning of the chromatogram. TEA is essentially non–polar at acidic pH, so SPE should be carried out at low pH, allowing TEA to be well retained on the cartridge. TEA is essentially polar at an alkaline pH, and so elutes earlier on the chromatogram, where fewer matrix constituents elute in the background after SPE cleanup. This improves the performance characteristics of TEA detection and quantification. However, the reversed–phase SPE can accumulate those non–polar matrix solutes, which typically co–elute with the lipophilic target compounds, and thus cause ion suppression. Purification employing SPE can be improved using an orthogonal separation by applying anion ion exchange cartridges, but this then excludes TEN analysis that has no retention on such cartridges. 

In our study, we applied sample dilution to reduce the concentration of background compounds. After a 5–fold sample dilution, the MEs still influenced the analysis, mainly in flour samples, and hence the application of IDMS was inevitable. If IDMS is not available, the time–consuming and more complicated matrix–matched calibration could be used, but this quantification approach failed for AME and TEA during interlaboratory comparisons [10,13,31]. The use of SPE is necessary if all five toxins need to be analyzed at the desired levels (down to 1 µg/kg) set by CEN. Our study is the first to detect five toxins at LOQs lower than 1.0 µg/kg in tomato–based and flour samples without applying SPE. Sunflower seed–based samples were not tested with our method. Thus far, we focused only on tomato and flour matrices. Due to the complexity of sunflower seeds, mainly the unpeeled ones, the dilute and shoot approach may not allow such low LOQs in tomato and flour samples. Those studies, which also excluded the purification steps, used mainly acetonitrile–based extractions (Table S1). The acetonitrile–based extraction in combination with the QuECHERS approach is straightforward and beneficial if other contaminants (i.e., pesticides) need to be determined in a food extract. 

### 3.2. Method Application

The method is currently under accreditation. Future studies may include a survey of tomato and wheat samples after receiving the accredited status of the developed method. This quick method will be used in a foreseen project in which food samples are treated with the ozone to degrade the toxins. Such investigations need fast analysis carried out using the present method.

## 4. Materials and Methods 

### 4.1. Standards, Reagents, Equipment, and Samples

Dried analytical standards (100 µg) were obtained from Romer Labs (Tulln, Austria). Stock solutions were prepared by adding 1.0 mL methanol to the vial and standards were re–dissolved. Stock solutions were kept at –18 °C for a year. The isotopically labeled analogs (ISTDs) were purchased from Angewandte Synthesechemie Adlershof (ASCA) GmbH (Berlin, Germany). The same ISTD mixture was prepared as recommended by JRC. It contained AOH–d3 (0.5 µg/mL), AME–d3 (0.5 µg/mL), TEA–^13^C2 (2.5 µg/mL), TEN–d3 (0.5 µg/mL), and ALT–d6 (1 µg/mL) in methanol and stored at –18 °C for half of a year. Methanol, ammonia (25%), acetic acid, and ammonium acetate (either LC–MS or HPLC grade), Ascentis Express HPLC column (100 mm × 3 mm, 2.7 µm) were purchased from the Merck–Sigma group (Schnelldorf, Germany). The hydrophilic PTFE syringe filters (13 mm, 0.45 µm), as well as the Phenomenex HPLC pre–column holder and cartridge (4 mm × 3 mm), were obtained from Gen–lab Ltd. (Budapest, Hungary). The LC–MS/MS analysis was carried out using a Shimadzu Nexera LC–30AD liquid chromatograph, consisting of a SIL–30AC auto sampler, CTO–20AC column oven, and CBM–20A communications bus module (Shimadzu Corporation, Kyoto, Japan), which was coupled to an AB Sciex 6500+ QTRAP triple quad MS detector and equipped with an IonDrive Turbo V Source (Sciex; Warrington, Cheshire, UK). Data acquisition and evaluation were performed using Analyst software version 1.7.1 and MultiQuant software version 3.0.3, respectively (Sciex; Warrington, Cheshire, UK). Sample shaking and centrifugation were done using a horizontal shaker (SM 30 B; Edmund Bühler, Bodelshausen, Germany) and a Jouan B4i centrifuge (Thermo Fisher Scientific, Budapest, Hungary), respectively. Sample evaporation was carried out using a TurboVap II (Biotage, Uppsala, Sweden). The naturally contaminated or spiked tomato puree and wheat quality check (QC) samples (P49, N22, R61, Y21, H60, B56, G28) used in our study were leftovers from the MVS investigation, organized by JRC in 2018 and stored at −18 °C until subjected to analysis.

### 4.2. Sample Preparation

Flour samples required appropriate milling to obtain a particle size not higher than 1.0 mm. In our study, the degree of grind was 0.5 mm in order to obtain a homogenous sample. Further, 2.00 g of the sample could be used for extraction.

Samples (2.00 g) were weighed in 50.0 mL polypropylene centrifuge tubes (Figure S1). Tomato–based samples (tomato puree, ketchup, and tomato sauce) were extracted with 9.0 mL of methanol. The flour samples (wheat, rye, and maize) were extracted with 10.0 mL of a methanol–water–acetic acid (80/19/1, *v*/*v*/*v*) mixture [10]. After adding the extraction solvent to the samples, the tubes were capped, vortex–mixed, and shaken for 1 h. Afterwards, the tubes were centrifuged at an ambient temperature for 10 min at 2800 g and 1.0 mL upper layers were collected in glass evaporating tubes containing 10 µL of the ISTD solution. Samples were evaporated to dryness at 50 °C under a gentle stream of nitrogen. Sample residues were reconstituted in 300 µL of methanol by vortex–mixing for 10 s. Then, 700 µL water was added into the tubes to obtain a total volume of 1.00 mL volume, followed by vortex–mixing for an additional 10 s. It is important to re–dissolve the sample in pure methanol first, followed by adding water. This leads to total reconstitution of toxins in the injected solution. This solvent change was necessary to avoid the deformation of the chromatographic peak of TEA caused by a different eluotropic strength of the initial mobile phase composition and the extraction medium. Finally, samples were filtered through hydrophilic PTFE syringe filters (0.45 µm, 13 mm) into HPLC vials. This preparation resulted in 5–fold diluted samples for each matrix [10]. For those samples containing toxin concentrations above the calibration curve, the extracts (100 µL) were diluted with 890 µL water in glass tubes. Afterwards, the ISTD solution (10 µL) was added to the diluted extracts, followed by vortex–mixing and syringe filtration into HPLC vials.

### 4.3. LC–IDMS Determination

Toxins were separated using an alkaline mobile phase and a binary gradient elution mode. Solvent A consisted of 5 mM ammonium acetate in water (pH adjusted between 8.0 and 8.8 with ammonia) and solvent B consisted of methanol. Retention time shifts between pH 8.0 and 8.8 were not observed. The mobile phase gradient consisted of 10% B at 0 min; 10% B at 1.0 min; 95% B at 8.0 min; 95% B at 12.0 min; and 10% B at 12.1 min. The stop time was 15.0 min. An equilibration time of 3.0 min was adequate due to the low dwell volume of the UPLC system. This was supported by no shift in retention time between injections. The column thermostat and auto sampler were maintained at 25 °C. The flow rate was 0.45 mL/min. The injection volume was 10.0 µL. Compounds were detected by employing a negative ion mode and multiple reaction monitoring (MRM) scan mode. The ion source parameters were as follows: curtain gas 40 unit, gas1 40 unit, gas2 40 unit, drying gas temperature 350 °C, and interface heater on. The higher temperature and flow of drying gases did not improve the sensitivity. Ion transitions are listed in Table 1.

### 4.4. Quantification

A five–point calibration curve was prepared in water that met the requirement of the candidate method [10]. The calibrants contained 10.0 µL ISTD solution and had a final volume of 1.00 mL. The levels for AOH, AME, and ALT were between 0.1 ng/mL and 10 ng/mL (corresponding to 0.5–50 µg/kg mass fraction in sample). In the case of TEA, the calibration was between 1.0 ng/mL and 100 ng/mL (5.0–500 µg/kg). For TEN, the calibration was set from 0.5 ng/mL to 50.0 ng/mL (2.5–250 µg/kg). The linear function between concentrations and isotope ratios was fit–for–purpose in these concentration ranges, and the determination coefficient was not lower than 0.9950.

In the LC–IDMS analysis, the isotope ratio was measured, as it is the response ratio of analyte to ISTD. The isotope ratios are plotted against the concentrations to obtain the calibration curve. Since the analyte and its corresponding ISTD co–elute, the ISTD undergoes the same effects in the ion source as the analyte. Consequently, the isotope ratio will be independent on the matrix effect, at least up to a certain degree. In this case, there is no need to prepare the matrix–matched calibration to compensate the matrix effect.

## 5. Conclusions 

A dilute and shoot method was developed in order to analyze *Alternaria* toxins. The method was validated for tomato–based samples and different flours using both spiked and naturally contaminated samples. The matrix effect was thoroughly studied and we observed that the sample dilution lowered the matrix effect. The approach enabled quick sample preparation and accurate quantification based on isotope dilution. A limitation of the presented strategy could be the need for the high sensitivity LC–MS/MS instrument. Moreover, the method was not tested for unpeeled sunflower seeds, which contained a much more complex sample matrix. Its analysis was recommended by CEN.

## Figures and Tables

**Table 1:** Tandem mass spectrometric (MS/MS) detection parameters for *Alternaria* toxins optimized for AB Sciex 6500+ QTRAP instrument equipped with IonDrive Turbo V Source.

Compounds	Precursor Ion (*m/z*)	Product Ion (*m/z*)	Dwell Time (ms)	DP (V)	EP (V)	CE (V)	CXP (V)	IR% in Solvent	IR% in Samples	PTL for Ion Ratio% (± 30%)
AOH	257.1	**215.1**	50	−60	−10	−35	−15	73	69–80	51–95
		212.1	50			−35	−8			
AOH-d3	260.1	**218.1**	50	−60	−10	−35	−15	-	–	–
AME	271.1	**256.1**	50	−60	−10	−27	−16	23	17–24	16–30
		228.1	50			−36	−18			
AME-d3	274.1	**259.1**	50	−60	−10	−36	−16	-	–	–
TEA	196.0	**139.0**	50	−50	−10	−27	−9	64	60–72	45–83
		112.0	50			−30	−9			
TEA-^13^C2	198.0	**141.1**	50	−50	−10	−27	−9	-	–	–
TEN	413.2	**141.1**	50	−70	−10	−25	−7	72	67–78	50–94
		271.1	50			−22	−16			
TEN-d3	416.2	**274.1**	50	−70	−10	−22	−16	-	–	–
ALT	291.1	**214.1**	50	−75	−10	−29	−15	66	50–74	46–86
		186.1	50			−35	−10			
ALT-d6	296.1	**189.1**	50	−75	−10	−35	−10	-	-	-

AOH: Alternariol; AME: Alternariol monomethyl ether; TEA: Tenuazonic acid; TEN: Tentoxin; ALT: Altenuene; DP: declustering potential; EP: entrance potential; CE: collision energy; CXP: collision cell exit potential; IR: ion ratio; PTL: permitted tolerance limit. The quantification ion transitions are highlighted with bold.

**Table 2:** Results of the quality check (QC) sample analysis using the dilute and shoot method. The reference values and corresponding standard deviations, obtained from the method validation trial, are in brackets below the detected concentration.

Sample Code	Matrix	Detected Concentration	Trueness%	Detected Concentration	Trueness %	Detected Concentration	Trueness %	Detected Concentration	Trueness %	Detected Concentration	Trueness %
		AOH		AME		TEA		TEN		ALT	
P49	Naturally contaminated tomato puree	25.6 µg/kg (27.4 ± 3.45)	93.4	14.8 µg/kg (16.4 ± 1.89)	90.2	997 µg/kg (961 ± 58.6)	104	-	-	-	-
N22	Naturally contaminated tomato puree	11.7 µg/kg (12.9 ± 2.01)	90.7	5.26 µg/kg (6.19 ± 0.68)	85.0	460 µg/kg (499 ± 39.8)	92.2	-	-	-	-
R61	Naturally contaminated tomato puree	5.88 µg/kg (6.06 ± 0.86)	97.0	2.34 µg/kg (2.69 ± 0.32)	87.0	182 µg/kg (183 ± 11.5)	99.4	-	-	-	-
Y21	Spiked tomato puree	1.39 µg/kg (1.82 ± 0.26)	76.4	1.45 µg/kg (1.98 ± 0.26)	73.2	45.1 µg/kg (47.0 ± 4.88)	96.0	50.1 µg/kg (44.9 ± 4.21)	111	1.69 µg/kg (2.18 ± 0.41)	77.5
H60	Spiked tomato puree	9.16 µg/kg (9.68 ± 1.33)	94.6	8.56 µg/kg (9.74 ± 0.86)	87.9	201 µg/kg (194 ± 16.1)	104	225 µg/kg (218 ± 34.4)	103	10.1 µg/kg (11.2 ± 1.86)	90.2
B56	Naturally contaminated wheat	1.54 µg/kg (2.04, not evaluated in the trial)	75.5	0.63 µg/kg (0.55, not evaluated in the trial)	115	282 µg/kg (265 ± 19.7)	106	55.7 µg/kg (52.2 ± 6.68)	107	-	-
G28	Naturally contaminated wheat	1.52 µg/kg (1.83 ± 0.50)	83.1	0.68 µg/kg (1.29 ± 0.34)	52.7	145 µg/kg (162 ± 14.4)	89.5	3.44 µg/kg (5.29 ± 1.31)	65.0	-	-

**Table 3:** Validation results for *Alternaria* toxins in tomato-based products and in flour using the dilute and shoot method. Matrix effect (ME) was evaluated for both matrices using the ESTD (external standard) and ISTD (internal standard) methods.

Compound	AOH	AME	TEA	TEN	ALT
	Tomato				
Absolute recovery% (n = 10)	66.7–75.4	67.6–74.6	75.7–91.6	87.3–88.7	82.9–89.0
Precision (RSD%)	2.8–6.3	1.0–3.5	1.7–2.5	2.6–5.5	4.1–7.6
Absolute ME% (ESTD)	23.8–48.7 ion suppression	0.4–6.8 ion suppression	0.6–4.9 ion suppression	0.8–7.2 ion suppression	6.6–7.7 ion enhancement
Relative ME% (ESTD)	20.8	4.8	4.5	6.4	0.32
Absolute ME% (ISTD)	4.9–9.7 ion suppression	0.5–7.3 ion suppression	2.0–6.5 ion suppression	1.3–3.7 ion suppression	2.6–7.5 ion suppression
Relative ME% (ISTD)	3.2	5.2	4.1	2.7	0.56
LOQ (µg/kg)	0.20	0.02	0.50	0.10	0.50
	Flour				
Absolute recovery% (n = 10)	69.0–76.3	68.1–75.1	77.7–90.0	87.8–89.2	84.5–89.0
Precision (RSD%)	3.2–7.7	1.9–4.9	0.8–2.8	3.5–4.6	7.7–8.3
Absolute ME% (ESTD)	62.0–53.9 ion suppression	18.4–25.6 ion suppression	0.1–10.3 ion suppression	21.6–35.8 ion suppression	48.3–66.3 ion suppression
Relative ME% (ESTD)	10.1	5.0	5.1	10.4	6.8
Absolute ME% (ISTD)	2.3–6.8 ion suppression	1.0–4.1 ion suppression	0.7–1.4 ion suppression	0.2–5.9 ion suppression	5.4–9.9 ion suppression
Relative ME% (ISTD)	5.2	2.6	0.4	3.0	2.6
LOQ (µg/kg)	0.20	0.04	0.70	0.15	0.80

RSD: relative standard deviation; ISTD: internal standard; ESTD: external standard.

## Data Availability

The data presented in this study are available on request from the corresponding author.

## Supplementary Materials

Table S1: Existing LC–MS/MS methods reporting *Alternaria* toxin determination in tomato– and cereal–based samples. Figure S1: Flow chart of the dilute and shoot strategy for analyzing *Alternaria* toxins.
