# Supplementary material for: A Dilute and Shoot Strategy for Determining Alternaria Toxins in Tomato-Based Samples and in Different Flours Using LC-IDMS Separation
Source: Molecules. 2021 Feb 15;26(4):1017. doi: 10.3390/molecules26041017 (PMC7918963; doi:10.3390/molecules26041017)

Table S1: Existing LC-MS/MS methods reporting *Alternaria* toxin determination in tomato and cereal based samples.

Compounds	Matrix	Method	Isotope dilution	Extraction solvent	Clean-up	Analytical limit	Reference
AOH, AME, TEA, TEN, ALT	Tomato based products, cereals, sunflower seeds	LC-ESI-MS/MS, negative ionization, C-18 column, pH 8	TEA-^{13}C2, ALT-d6, AOH-d3, TEN-d3, AME-d3	MeOH/water/acetic acid, 84/15/1, (v/v/v)	SPE	LOQ: 0.19 – 2.17 µg/kg	[13]
AOH, AME, TEA, TEN, ALT	Cereals method1	LC-ESI-MS/MS polarity switching, HSS-T3 column, pH 3	No	AcN + 1% (v/v) acetic acid	Not performed	LOQ: 2 µg/kg	[14]
AOH, AME, TEA, TEN, ALT	Wine, beverages, cereals method2	LC-ESI-MS/MS polarity switching, HSS-T3 column, pH 3	No	AcN/water, 84/16 (v/v) + 1% formic acid	Not performed	LOQ: 1.0-5.0 µg/kg	[14]
AOH, AME, TEA, TEN, ALT	Sunflower oil, sunflower seeds, tomato based foodstuff	LC-ESI-MS/MS polarity switching, HSS-T3 column, pH 3	No	AcN/water, 84/16 (v/v) + 1% formic acid	Not performed	LOQ: 1.5-5.0 µg/kg	[15]
AOH, AME, TEA, TEN, ALT and other toxins	Tomato products, bakery products, sunflower seeds, fruit juices, and vegetable oils	LC-ESI-MS/MS, polarity switching, RP-Amide column, pH 3	No	AcN/water/formic acid, ratio depends on the matrix of interest	Not performed	LOQ: 0.1 - 110 µg/kg	[16]
AOH, AME, TEA, TEN, ALT	Tomato	LC-ESI-MS/MS, negative ionization, chemical derivatization, C-18 column, pH 3	No	MeOH	SPE	LOQ: 2-20 µg/kg	[17]

TEN, AOH, AME	cereal based foodstuff, sunflower seed, tomato-based foodstuff	LC-ESI-MS/MS, positive ionization, C-18 column, pH 7	AOH-^{13}C14 AME-^{13}C15 TEN-d3	AcN/water 84/16 + pentane for defatting	Not performed	LOQ: 0.27-1.16 µg/kg	[18]
TEA	Cereals	LC-ESI-MS/MS, positive ionization, chemical derivatization, C-18 column, pH 3	No	Ethyl acetate	Not performed	LOQ: 50 µg/kg	[19]
AOH, AME, TEA, TEN, ALT, conjugates, other toxins	Tomato, cereals	LC-ESI-MS/MS, negative ionization, C-18 column, pH 8.7	No	MeOH/water/acetic acid, 79/20/1 (v/v/v) hexane	Not performed	LOQ: 0.06 – 12.0 µg/kg	[20]
AOH, AME, TEA, TEN	Cereals, tomato and fruit based samples	LC-ESI-MS/MS, negative ionization, C-18 column, pH 7	TEA-^{13}C6-^{15}N, AOH-d4, AME-d4	ACN/water, 84/16 (v/v) + 1,3% formic acid	SPE	LOQ: 0.02 - 5.56 µg/kg	[21]
AOH, AME, TEA, TEN	tomato and fruit based samples	LC-ESI-MS/MS positive ionization, phenyl-hexyl column	No	AcN + 1% (v/v) formic acid	QuEChERS	LOQ: 4.0 - 160 µg/kg	[22]
AOH, AME, TEA, TEN	Vegetables, fruits	LC-ESI-MS/MS polarity switching, C-18 column, pH 7	No	AcN + 1% (v/v) formic acid	QuEChERS	LOQ: 0.2 – 10 µg/kg	[23]
AOH, AME, TEA, TEN, ALT, conjugates	Tomato, fruit, vegetables	LC-ESI-MS/MS, polarity switching, HSS T3 column, pH 3	TEA-^{13}C6-^{15}N, AME-d4	AcN	QuEChERS	LOQ: 1.1 – 61.5 µg/kg	[24]

AOH, AME, TEA, TEN, ALT, conjugates	cereal-based foodstuffs	LC-ESI-MS/MS, polarity switching, HSS T3 column, pH 3	TEA-$^{13}C6$-^{15}N, AME-d4	AcN/water/acetic acid, 79/19.5/1.5, (v/v/v)	Not performed	LOQ: 0.9 – 8.3 µg/kg	[25]
AOH, AME, TEA, TEN, ALT and other toxins	wine, vegetable juices and fruit juices	LC-ESI-MS/MS, negative ionization, C-18 column, pH 9	No	Dilution with sodium hydrogen carbonate buffer, pH 8.4	SPE	LOQ: 0.4 – 1.9 µg/kg	[26]
AOH, AME	Tomato based product	LC-ESI-MS/MS, negative ionization, HSS T-3 column, pH 2	AOH-d4, AME-d4	AcN	MIP	LOQ: 0.6 – 4.6 µg/kg	[27]
AOH, AME, TEN	Tomato, tomato based products	LC-ESI-MS/MS, positive ionization, C-18 column, pH 3	No	AcN	Dispersive liquid-liquid microextraction	LOQ: 1.8 - 3.5 µg/kg	[28]
AOH, AME, TEA, TEN	Tomato, ketchup, fruit juices	LC-ESI-MS/MS, negative ionization, C-18 column, pH 8.8	No	AcN/water/MeOH, 45/45/10, (v/v/v)	SPE	LOQ: 0.3 - 20 µg/kg	[29]
AOH, AME, TEA, TEN, ALT	Selected Food Commodities	LC-ESI-MS/MS, negative ionization, C-18 column, pH 8	TEA-$^{13}C2$, ALT-d6, AOH-d3, TEN-d3, AME-d3	AcN + 0.1% (v/v) formic acid	QuEChERS, defatting with hexane	LOQ: 0.5 - 10 µg/kg	[30]
TEA, AOH, TEN, AME	Korean barley grain samples, rice culture medium extract	LC-ESI-MS/MS, positive ionization, C-8 column, pH 3	No	MeOH	Not performed	LOQ: 0.25 - 8.0 µg/kg	[32]
ALT, AOH, AME,	Sorghum	LC-ESI-MS/MS, positive ionization, C-18 column, pH 3	No	MeOH/ethyl acetate/water, 70/20/10, (v/v/v)	SPE	LOQ: 6.0 – 25 µg/kg	[33]

AOH, AME, TEA, TEN, ALT	Beverages, tomato, basil, olive	LC-ESI-MS/MS, polarity switching, C-18 column, pH 7	No	MeOH	SPE	LOQ: 0.7 - 63 µg/kg	[34]
TEA	Tomato based foodstuff	LC-ESI-MS/MS, negative ionization, chemical derivatization, C-18 column, pH 7	TEA-$^{13}C6$-^{15}N	Ethyl acetate	SPE	LOQ: 0.3 µg/kg	[35]
AOH, AME, TEA, TEN, ALT, conjugates, other toxins	Tomato sauce, wheat flour and sunflower seed oil	LC-ESI-MS/MS, negative ionization, C-18 column, pH 8.7	No	MeOH/water/acetic acid, 79/20/1 (v/v/v) hexane	Not performed	LOQ: 0.06 – 12.0 µg/kg	[36]
AOH, AME, TEA, TEN, ALT	Tomato based samples, flour	LC-ESI-MS/MS, negative ionization, C-18 column, pH 8-8.8	TEA-$^{13}C2$, ALT-d6, AOH-d3, TEN-d3, AME-d3	MeOH/water/acetic acid, 84/15/1, (v/v/v)	Not performed	LOQ: 0.02 – 0.5 µg/kg	Present study

TEA: tenuazonic acid; ALT: altenuene; AOH: alternariol; TEN: tentoxin; AME: alternariol monomethyl ether; AcN: acetonitrile; MeOH: methanol; MIP: molecularly imprinted polymer; LOQ: limit of quantification; LOD: limit of detection; SPE: solid phase extraction; QuEChERS: quick, easy, cheap, effective, rugged, and safe

Figure S1: Flow chart of the dilute and shoot strategy for analyzing *Alternaria* toxins.

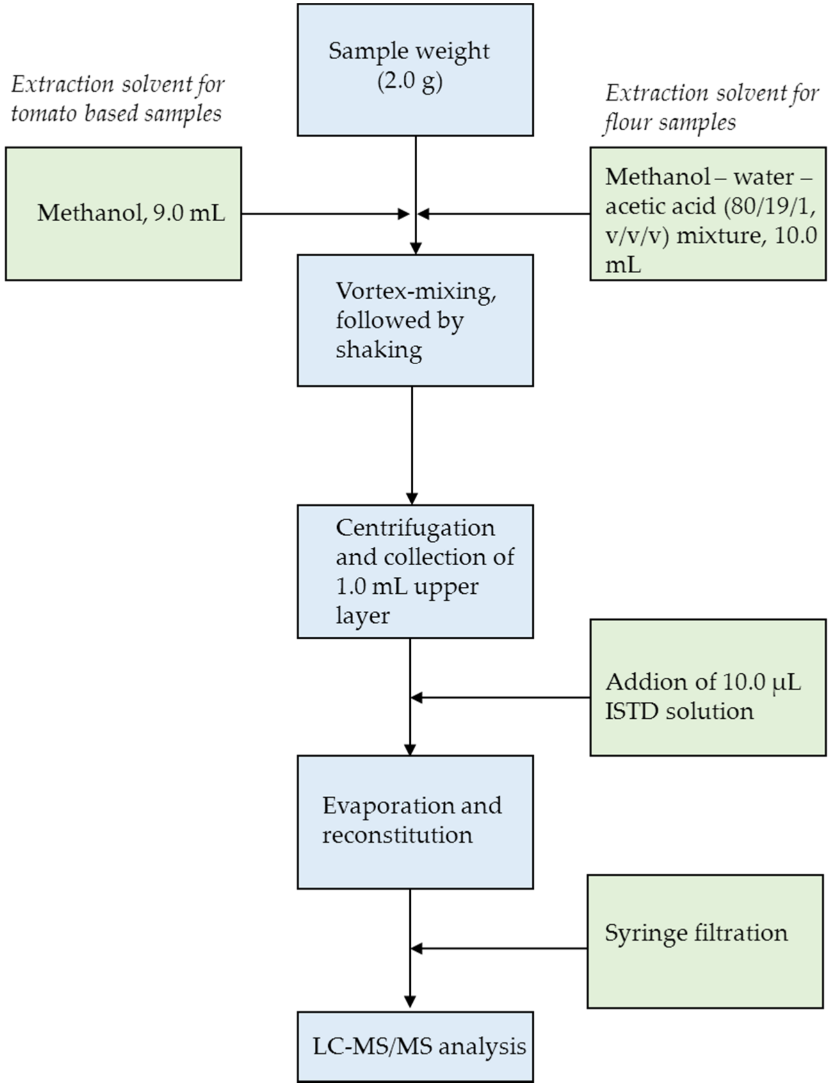

Supplement: Supplementary file 1 [file molecules-26-01017-s001.pdf]
